# Practical measures for sustainable shark fisheries: Lessons learned from an Indonesian targeted shark fishery

**DOI:** 10.1371/journal.pone.0206437

**Published:** 2018-11-02

**Authors:** Irfan Yulianto, Hollie Booth, Prayekti Ningtias, Tasrif Kartawijaya, Juan Santos, Sarmintohadi, Sonja Kleinertz, Stuart J. Campbell, Harry W. Palm, Cornelius Hammer

**Affiliations:** 1 Wildlife Conservation Society Indonesia Program, Bogor, Indonesia; 2 Faculty of Fisheries and Marine Sciences, Bogor Agricultural University, Bogor, Indonesia; 3 Institute of Baltic Sea Fisheries, Thuenen Institut, Rostock, Germany; 4 Directorate of Marine Conservation Areas and Biodiversity, Ministry of Marine Affairs and Fisheries, Jakarta, Indonesia; 5 Department of Aquaculture and Sea-Ranching, University of Rostock, Rostock, Germany; 6 Rare Indonesia, Bogor, Indonesia; University of Bremen, GERMANY

## Abstract

Overfishing is a major threat to the survival of shark species, primarily driven by international trade in high-value fins, as well as meat, liver oil, skin and cartilage. The Convention on the International Trade in Endangered Species of Wild Fauna and Flora (CITES) aims to ensure that commercial trade does not threaten wild species, and several shark species have recently been listed on CITES as part of international efforts to ensure that trade does not threaten their survival. However, as international trade regulations alone will be insufficient to reduce overexploitation of sharks, they must be accompanied by practical fisheries management measures to reduce fishing mortality. To examine which management measures might be practical in the context of a targeted shark fishery, we collected data from 52 vessels across 595 fishing trips from January 2014 to December 2015 at Tanjung Luar fishing port in East Lombok, Indonesia. We recorded 11,920 landed individuals across 42 species, a high proportion of which were threatened and regulated species. Catch per unit effort depended primarily on the number of hooks and type of fishing gear used, and to a lesser degree on month, boat engine power, number of sets and fishing ground. The most significant factors influencing the likelihood of catching threatened and regulated species were month, fishing ground, engine power and hook number. We observed significant negative relationships between standardised catch per unit effort and several indicators of fishing effort, suggesting diminishing returns above relatively low levels of fishing effort. Our results suggest that management measures focusing on fishing effort controls, gear restrictions and modifications and spatiotemporal closures could have significant benefits for the conservation of shark species, and may help to improve the overall sustainability of the Tanjung Luar shark fishery. These management measures may also be applicable to shark fisheries in other parts of Indonesia and beyond, as sharks increasingly become the focus of global conservation efforts.

## Introduction

Overfishing is the greatest global threat to marine fish stocks [1–5]. Several shark species (Selachimorpha) are particularly vulnerable to overexploitation due to their conservative life history strategies, large body sizes and the high economic value of their preserved body parts [6–8]. With increasing fishing pressure in recent decades, primarily driven by international demand for a range of consumer goods (including fins, liver oil, skin, cartilage and meat), it is estimated that annual fishing mortality now exceeds the intrinsic rebound potential of most commercially exploited species [5, 9, 10]. This fishing pressure is taking its toll, with an estimated one in four Chondrichthyan species now threatened with extinction, making sharks amongst the most threatened species groups in the world [11].

It is also increasingly acknowledged that sharks play a critical role in maintaining functional and productive ocean ecosystems [7], as well as providing an important source of food and income for many coastal communities [12]. Recognising both the plight and importance of shark populations, there is growing professional and public interest to improve shark conservation, and the management of shark fisheries and trade [13]. This is reflected in several recent policy decisions to afford new international regulations for 12 species of sharks across seven genera under the Convention on the International Trade of Endangered Species of Wild Fauna and Flora (CITES). This is a promising step for shark conservation; however, in order to create tangible outcomes for species conservation CITES must be implemented through domestic measures that are adapted to national and local contexts.

Indonesia is the world’s largest shark fishing nation [9, 14], and a global priority for shark conservation [15]. Until recently Indonesia’s shark fishery has largely functioned as de facto open-access [12, 16]. However, in the past five years the Indonesian government has demonstrated a clear commitment to shark conservation and resource management, with domestic measures put in place to implement international obligations under CITES [17]. Exploitation of all CITES-listed species is now regulated, either through full species protection or export controls (these species are hereafter referred to as ‘regulated’ species). However, CITES only affords protection to a small number of Indonesia’s 112 known shark species [18], of which 83 are threatened with extinction according to the IUCN Red List of Threatened Species (i.e. Vulnerable (VU), Endangered (EN) or Critically Endangered (CR) [19], these species are hereafter referred to as ‘threatened’ species), many of which continue to be landed throughout the country [20]. Further, these policy measures predominantly regulate trade at the point of export, but do not necessarily influence fisher behaviour or local demand at the point of catch, such that the ‘trickle-down’ impacts on species mortality are unknown. In addition, effectively implementing species-specific shark mortality controls remains challenging due to the non-selectivity of fishing gears, and practical and cultural barriers to changing fisher preferences for certain gear-types and fishing methods. As such, existing regulations alone (e.g. Indonesian Law on Fisheries 31/2004 and its derivative regulations) will likely be insufficient to curb mortality of threatened and regulated species, as fishers must be both willing and able to change their fishing behaviour [21]. Moreover, most of Indonesia’s shark fisheries are small-scale, and in relatively poor coastal communities where there are often no legal, sustainable marine-based alternatives to shark fishing that offer similar financial returns [22, 23]. It is therefore imperative to consider the ethical and socioeconomic impacts of shark trade controls. Most shark species listed under CITES are listed on Appendix II, which is designed for sustainable use. International trade is permitted for CITES Appendix II species provided it is non-detrimental to wild populations of the species, as proven through a scientific non-detriment finding (NDF) report and implemented through a system of export permits. However, in Indonesia there is currently a lack of species-specific trade data for conducting NDFs and setting sustainable export quotas, such that the Indonesian government has to introduce trade bans for these species in order to meet CITES obligations. With new CITES-listings for thresher sharks (*Alopias* spp.) and silky shark (*Carcharhinus falciformis)* recently coming into force, this is likely to have huge implications for Indonesia’s economically important shark industry, and the coastal communities depending on it. In order to balance conservation and socioeconomic objectives, robust management systems must be put in place that ensure and allow sustainable fishing and trade. This necessitates the identification of practical management measures that can reduce mortality of threatened and regulated species at the point of catch, and provide realistic options for fishers to effectively and measurably improve the sustainability of their fishing practices.

This study analyses two years of qualitative and quantitative data from one of Indonesia’s targeted shark fisheries in Tanjung Luar, West Nusa Tenggara Province. We outline the key characteristics of the fishery, including fishing behaviour and overall catch volumes and composition. We analyse the impacts of different fishing techniques, and present factors influencing overall catch per unit effort (CPUE) of individual shark fishing trips, as well as factors influencing the likelihood of catching threatened and regulated species. Finally, we discuss the implications of our findings, and provide practical recommendations for fisheries management measures, which can support CITES implementation for sharks and reduce the catch of threatened and regulated species, in Indonesia and beyond.

## Methods

This work was conducted under a Memorandum of Understanding (MoU) and Technical Cooperation Agreement (TCA) between the Wildlife Conservation Society (WCS) and the Ministry of Environment and Forestry (MoEF), Ministry Marine Affairs and Fisheries (MMAF) and the Marine and Fisheries Agency (MFA) of West Nusa Tenggara Province. These documents were approved and signed by Sonny Partono (Director General of Conservation of Natural Resources and Ecosystem MoEF), Sjarief Widjaja (Secretary General MMAF), and Djoko Suprianto (Acting Head of MFA of West Nusa Tenggara Province). Due to this MoU and TCA no specific research permit was required. We collected data by measuring sharks that were already caught, dead, and landed by fishers in Tanjung Luar, with no incentives, compensation or specific requests for killing sharks for this study. WCS participates in the Conservation Initiative on Human Rights and the rules and guidelines of our Internal Review Board ensures that any research protects the rights of human subjects. We did not apply for an IRB permit for this study because our study design focused on collecting fish and fisheries data as opposed to personal socio-economic data. The FDGs and interviews were conducted to obtain early scoping information about fishing practices, and to establish protocols for more detailed fisheries data collection (as used in this study), and socio-economic data collection (as used in a later study (Lestari et al [23]), which underwent further ethical review due to the specific focus on human subjects).

### Site

**Fig 1:**
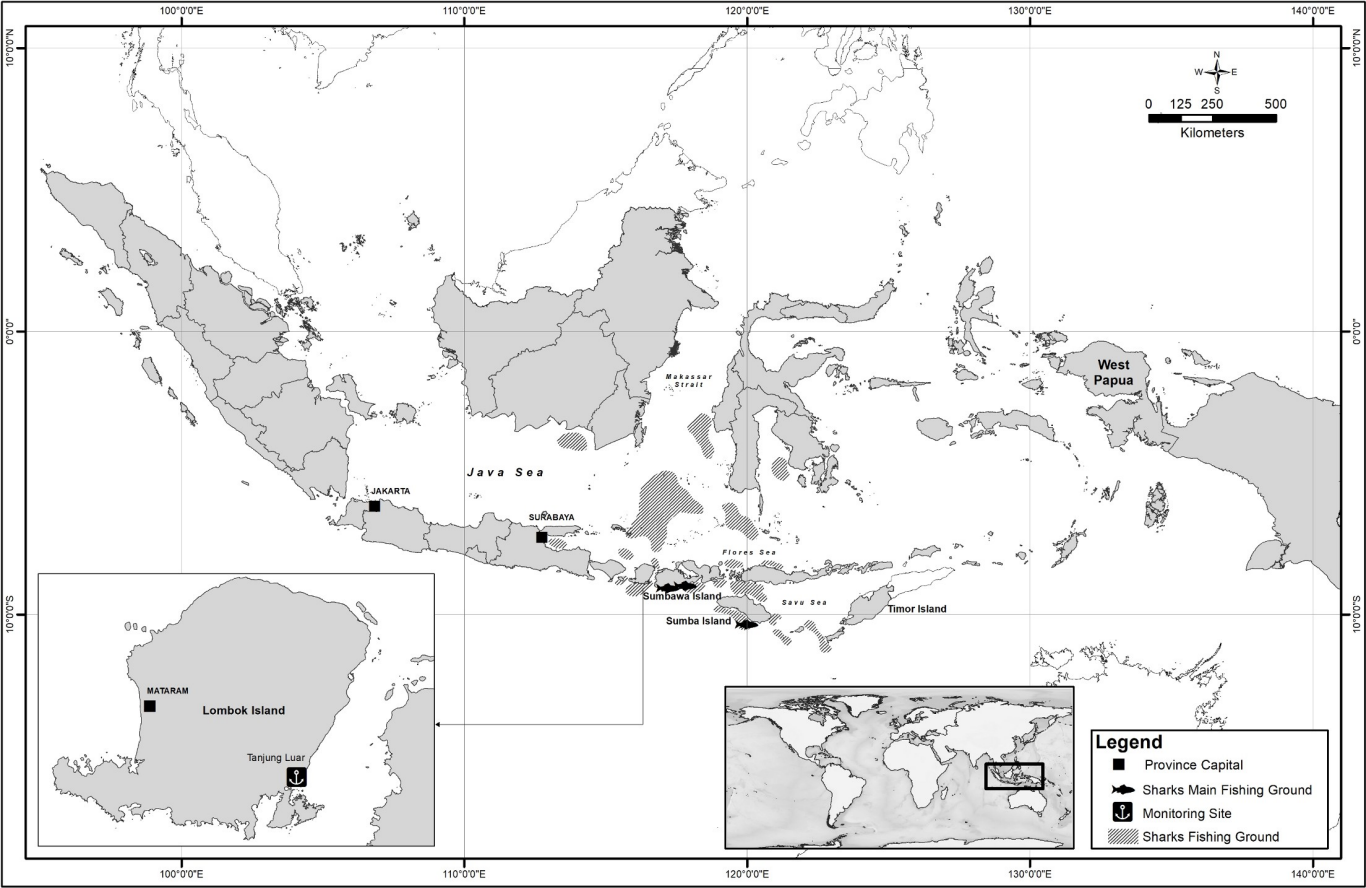
Sharks landing monitoring site and fishing grounds of shark fishers that land at Tanjung Luar.

Tanjung Luar, located in East Lombok, West Nusa Tenggara Province, Indonesia (Fig 1), is a landing site for one of Indonesia’s most well-known targeted shark fisheries. Tanjung Luar serves at least 1,000 vessels, and the majority of these are less than 10 gross tonnes (GT) in size [23]. A group of specialised fishers operating from Tanjung Luar village and a neighbouring island, Gili Maringkik, specifically target sharks. Shark catch is landed in a dedicated auction facility at the Tanjung Luar port. The shark industry is well established in Tanjung Luar, with product processing facilities and trade connections to local, national and international markets. Research by Lestari et al. [23] indicates that the shark industry is significantly more profitable than non-shark fisheries in Tanjung Luar, particularly for boat owners. Strong patron-client relationships exist between boat owners and fishers, with shark fishers exhibiting high dependency on shark fishing, limited occupational diversity and low adaptive capacity for shifting into other fisheries [23].

### Data collection

#### Qualitative data

In January 2014 we conducted preliminary scoping research to better understand the operational and socioeconomic characteristics of Tanjung Luar’s shark fishery. During a three-week scoping visit a team of four trained Indonesian enumerators conducted semi-structured interviews and focus group discussions (FDGs) with fishers, boat owners and traders, alongside naturalistic observation in the field. Respondents were selected through purposive sampling, since the research was exploratory in nature and *a priori* sampling decisions were not possible [24]. We conducted a total of 34 semi-structured interviews (S1 File) and four FDGs, which were attended by a total of 30 individuals. All interviews and discussions took place in Indonesian, with the help of a local enumerator who was fluent in the Tanjung Luar local dialect. Interviews took approximately 30 minutes, with no remuneration for participating. All respondents gave their full prior and informed consent before contributing to the research. During the interviews and FDGs we gathered information on number of boats, fishing gears used, fishing grounds, fishery operational characteristics, and shark supply chain, including estimated volumes and value of shark catch relative to other fisheries. We improved the accuracy of information on shark fishery characteristics and fishing behaviour through informal daily interactions and discussions with 131 shark fishers during our daily landings data collection and community engagement activities. More detailed socioeconomic data were collected in a full household survey in 2016, as outlined in Lestari et al. [23].

#### Quantitative data

Shark landings data were collected by three experienced enumerators, who were trained in species identification and data collection methods during a two-day workshop and three weeks of field mentoring to ensure the accuracy of the data collected. Landings were recorded every morning at the Tanjung Luar shark auction facility where shark fishers usually landed dead sharks, from 5am to 10am from January 2014 to December 2015. The enumerators recorded data on catch composition and fishing behaviour (Table 1) from 52 different vessels across a total of 595 fishing trips. The enumerators also measured the weight of selected sharks to calculate biomass and length-weight relationship.

**Table 1:** Types of data collected on fishing behaviour and catch composition during daily landings data collection at Tanjung Luar.

Item	Dataset	Type of data	Explanation and format
Fishing behaviour	Year	Categorical	Year that trip record was taken (2014–2015)
	Month	Categorical	Month that trip record was taken (Jan-Dec)
	Date	Categorical	Date that trip record was taken (1-28/30/31)
	Season	Categorical	Season (East / June—September, West / December—March, Transition I / April—May, Transition II / October—November)
	Engine power	Numeric	Engine horsepower
	Trip length	Numeric	Total number of days vessel was out at sea during trip
	Fishing ground	Categorical	Area of targeted shark fishing grounds (West Nusa Tenggara Province (WNTP), East Nusa Tenggara Province (ENTP), Other (fishing grounds outside of these province waters)
	Fishing gear	Categorical	Primary fishing gear used to target sharks (surface longline or bottom longline)
	Set	Numeric	Number of times primary fishing gear was deployed
	Hook number	Numeric	Number of hooks used per fishing trip
Catch composition	Length	Numeric	Total Length (TL) in cm
	Weight	Numeric	Total weight in kg (collected for selected individuals only)
	Sex	Categorical	Male/Female
	Species		Identification based on White et al. [47]

### Analysis

#### Catch volumes and composition

From fishing behaviour and catch data we calculated the overall species composition of catch. We calculated catch per unit effort (CPUE) by number of individuals using both catch per set (hereafter CPUE per set) and catch per 100 hooks per set (hereafter standardised CPUE) [25,26]. This was deemed necessary since different vessels and gear-types systematically deploy different numbers of hooks, and standardised CPUE allows for a more meaningful comparison.

#### Factors affecting CPUE

To understand factors influencing overall CPUE we log transformed CPUE per trip to fit a normal distribution, and fitted linear models (LMs) of CPUE per trip to fishing behaviour variables (Table 1). We considered all variables and used minimum AIC values with stepwise analysis of variance to identify the best fit and most significant influencing variables.

To inform the development of practical fisheries management measures (e.g. gear restrictions), we also specifically analysed differences in CPUE for surface and bottom longline gears employed in the fishery, using two-way ANOVAs.

#### Factors affecting catch of threatened and regulated species

To identify variables influencing the catch of threatened and regulated species we conducted a two-step process. In the first step, we identified factors influencing the likelihood of catching any threatened/regulated species during a given fishing trip, by creating binary response variables for whether a threatened species had been caught during a trip (yes = 1, no = 0), and separately for whether a regulated species had been caught during a trip (yes = 1, no = 0). We then fitted generalised linear models (GLMs) with binomial errors to the binary response variables, separately for catch of threatened species and catch of regulated species. In the second step we identified variables that significantly influenced the CPUE of threatened species and the CPUE of regulated species, given that any were caught. We removed all records in which no threatened or regulated species were caught, log transformed standardised CPUE of threatened and regulated species, and fitted linear models (LMs) of standardised CPUE of threatened species and standardised CPUE to regulated species to fishing behaviour variables. Again, we considered all meaningful models and used minimum AIC values with stepwise analysis of variance to identify the best fit [27] and most significant influencing variables. This approach was necessary since catch of threatened and regulated species is zero-inflated, and creating binary response variables with a binomial error structure allowed for a simpler and more powerful statistical analysis. Note that we conducted two separate analyses, one for threatened species only and one for regulated species only, but used the same methods and process, as outlined above, for each analysis. We did not group threatened and protected species together, since although some species are both threatened and protected, this is not the case for all shark species landed in Tanjung Luar.

## Results

### Fishery characteristics

A total of 52 shark fishing vessels operate from Tanjung Luar, all of which are classified as small-scale according to the Indonesian Ministry of Marine Affairs and Fisheries (MMAF) vessel categorisation system, with <7GT capacity. These vessels are operated by approximately 150 highly-specialised shark fishers, from Tanjung Luar village and Gili Maringkik, who make up roughly 5% of the local fisher population. The shark industry is more profitable than non-shark fisheries, and shark fishers report high household dependency on shark resources, low occupational diversity, and limited capacity and aspirations to move into other fisheries or industries.

Surface and bottom longlines are used as the primary fishing gears to target sharks, with pelagic fish (e.g. *Euthynnus* spp., *Rastrellinger* spp.) used as bait. Surface and bottom longlines systematically vary in length, depth deployed, number of sets, number of hooks used, and soak times (Table 2). Gear types are typically associated with certain vessel types, and fishers–captain and crew—tend to exhibit preferences for specific gear types. Shark fishers also use gillnets and troll lines as secondary gears, to catch bait and opportunistically target other species, such as grouper, snapper, skipjack and mackerel tuna.

**Table 2:** Characteristics of surface and bottom longlines.

Gear type	No. hooks	Deployment depth (m)	Length of mainline (m)	Length of branch line (m)	Distance between branch line (m)	Soak time (hours)	Typical vessel type
Surface longline	400–600	8–30	9,996–12,886	5–6	25–36	6	Larger vessel, >46 HP engine
Bottom longline	25–200	20–400	997–4,588	5–6	22–28	10	Smaller vessels <46HP engine

The shark fishing vessels can be divided into two broad categories according to fishing behaviour: larger vessels (≥14 m) with higher horsepower (HP) engines spend more time at sea than smaller vessels (≤12m) (p<0.001), and reach fishing grounds outside of West Nusa Tenggara. These vessels primarily fish in southern Sumbawa and Sumba Islands, however, they also reach as far as eastern Flores, Timor Island, and the Java Sea (Fig 1). Larger, higher HP vessels also tend to employ surface longlines (p<0.001), and since they spend more time at sea, have a higher number of sets per trip than smaller vessels (p<0.001). Smaller vessels (≤12 m) with smaller engines tend to remain in waters around West Nusa Tenggara only, carrying out shorter fishing trips using bottom longlines (Table 3).

**Table 3:** Characterisation of the different fishing vessels used to target sharks in Tanjung Luar.

Engine power (HP)	Engine type	Boat material	Boat size (m)	Boat crew (people)	Fishing gears	Number of boats (unit)	Fishing ground	Distance to fishing grounds (km)
46–60	Inboard	Wood	14–20	4–6	Surface/ bottom longlines	43	Sumba Island, south of Sumbawa, West Sumba and South Sumba, Savu Sea and Flores Sea, Java Sea, Makassar Strait	100–500
< 46	Inboard/ Outboard	Wood	7–12	2–4	Bottom longlines	9	Awang Bay, South of Kuta Lombok, Alas Strait, Panjang Island in Sumbawa	20–250

### Catch composition

During the study period we recorded shark catch from a total of 595 fishing trips. We recorded 11,678 individual sharks, with an average total catch of 963 individuals per month (SD ± 434) and 19.7 individuals per trip (SD ± 15.6). Standardised CPUE (per 100 hooks per set) ranged from 0.05 to 22.13 individuals, with an average of 0.96 and a mode of 0.20. Catch consisted of 42 different species from 18 families (Table 4). 22% of all landings were classified as threatened species (i.e. VU, EN, CR) according to the IUCN Red List of Threatened Species, and 73% were near threatened. Almost half (46.3%) of landings were regulated (i.e. CITES-listed) species. The most commonly caught species were silky shark (*Carcharhinus falciformis)*, black tip shark (*Carcharhinus limbatus)* and scalloped hammerhead (*Sphyrna lewini*).

**Table 4:** Sharks species landed in Tanjung Luar from January 2014 –December 2015 (VU = Vulnerable, EN = Endangered, NT = Near Threatened, LC = Least Concern, NE = Not Evaluated (VU and EN classified as ‘threatened’ in this study); II = CITES Appendix II, N = Not CITES-listed (II species classified as ‘regulated’ in this study)).

Family	Species	Common name	Number of sharks landed	Proportion of total catch (%)	Threatened (IUCN Red List Category) ^a^	Regulated (CITES listing) ^b^	Habitat	Environment
Alopiidae	*Alopias pelagicus*	Pelagic thresher	229	1.9%	VU	II	Pelagic	Oceanic
	*Alopias superciliosus*	Bigeye thresher	79	0.7%	VU	II	Pelagic	Oceanic
Carcharhinidae	*Carcharhinus albimarginatus*	Silvertip shark	145	1.2%	NT	N	Pelagic	Coastal
	*Carcharhinus amblyrhynchos*	Grey reef shark	13	0.1%	NT	N	Pelagic	Coastal
	*Carcharhinus brevipinna*	Spinner shark	95	0.8%	NT	N	Pelagic	Coastal
	*Carcharhinus falciformis*	Silky shark	3912	32.8%	NT	II	Pelagic	Oceanic
	*Carcharhinus plumbeus*	Sandbar shark	3	0.0%	VU	N	Pelagic	Coastal
	*Carcharhinus leucas*	Bull shark	16	0.1%	NT	N	Pelagic	Oceanic
	*Carcharhinus limbatus*	Black tip shark	2070	17.4%	NT	N	Pelagic	inshore/offshore
	*Carcharhinus longimanus*	Oceanic whitetip shark	3	0.0%	VU	II	Pelagic	Oceanic
	*Carcharhinus melanopterus*	Blacktip reef shark	27	0.2%	NT	N	Pelagic	Coastal
	*Carcharhinus obscurus*	Dusky whaler	378	3.2%	VU	N	Pelagic	Coastal
	*Carcharhinus sorrah*	Spot-tail shark	300	2.5%	NT	N	Pelagic	Coastal
	*Galeocerdo cuvier*	Tiger shark	985	8.3%	NT	N	Pelagic	Coastal
	*Negaprion acutidens*	Lemon shark^c^	1	0.0%	VU	N	Demersal	inshore/offshore
	*Prionace glauca*	Blue shark	949	8.0%	NT	N	Pelagic	Oceanic
	*Rhizoprionodon acutus*	Milk shark	3	0.0%	LC	N	Pelagic	inshore/offshore
	*Triaenodon obesus*	Whitetip reef shark	76	0.6%	NT	N	Pelagic	Coastal
Centorhinidae	*Cetorhinus maximus*	Basking shark	24	0.2%	VU	II	Pelagic	Coastal
Ginglymostomatidae	*Nebrius ferrugineus*	Tawny nurse shark	7	0.1%	VU	N	Pelagic	Coastal
Hemiscyllidae	*Chiloscyllium punctatum*	Brownbanded bambooshark	32	0.3%	NT	N	Pelagic	Coastal
Hexanchidae	*Heptranchias perlo*	Sharpnose sevengill shark	22	0.2%	NT	N	Demersal	inshore/offshore
	*Hexanchus griseus*	Bluntnose sixgill shark	4	0.0%	NT	N	Pelagic	inshore/offshore
	*Hexanchus nakamurai*	Bigeye sixgill shark	39	0.3%	NE	N	Demersal	inshore/offshore
Lamnidae	*Isurus oxyrinchus*	Shortfin mako	421	3.5%	VU	N	Pelagic	Oceanic
	*Isurus paucus*	Longfin mako	128	1.1%	VU	N	Pelagic	Oceanic
Orectolobidae	*Orectolobus leptolineatus*	Indonesian wobbegong	93	0.8%	NT	N	Pelagic	Coastal
Pseudotriakidae	*Pseudotriakis microdon*	False catshark	10	0.1%	NE	N	Demersal	Continental
Scyliorhinidae	*Atelomycterus marmoratus*	Coral catshark	10	0.1%	NT	N	Pelagic/ demersal	Coastal
Sphyrnidae	*Sphyrna lewini*	Scalloped hammerhead	1013	8.7%	EN	II	Pelagic	Coastal/semi oceanic
	*Sphyrna mokarran*	Great hammerhead	151	1.3%	EN	II	Pelagic	Coastal/semi oceanic
Squalidae	*Deania cf calcea*	Indonesian birdbeak dogfish^c^	7	0.1%	LC	N	Demersal	Continental
	*Squalus* cf *sp*.	NA	179	1.5%	-	N	Pelagic/ demersal	
	*Squalus* sp.1	NA	2	0.0%	-	N	Pelagic/ demersal	
	*Squalus* sp.2	NA	13	0.1%	-	N	Pelagic/ demersal	
Squatinidae	*Squantina* spp.	NA	7	0.1%	-	N	Demersal	
Stegostomatidae	*Stegostoma fasciatum*	Zebra shark	4	0.0%	EN	N	Pelagic	Coastal
Triakidae	*Hemitriakis* sp.	NA	221	1.9%	NE	N	Demersal	
	*Mustelus* cf *manazo*	Whitefin smoothhound	7	0.1%	NE	N	Demersal	Continental
**TOTAL**			**11,678**^d^					

^a^ IUCN Red List category as per December 2016, when analysis was conducted

^b^ CITES-listings as per December 2016, when analysis was conducted

^c^ These species were caught using gillnets only, and were therefore not included in the statistical analysis

^d^ Total catch using surface and bottom longlines was 11,569 individuals, the remaining 109 individuals were caught using gillnets, and were not included in the statistical analysis

### Factors affecting CPUE

**Fig 2:**
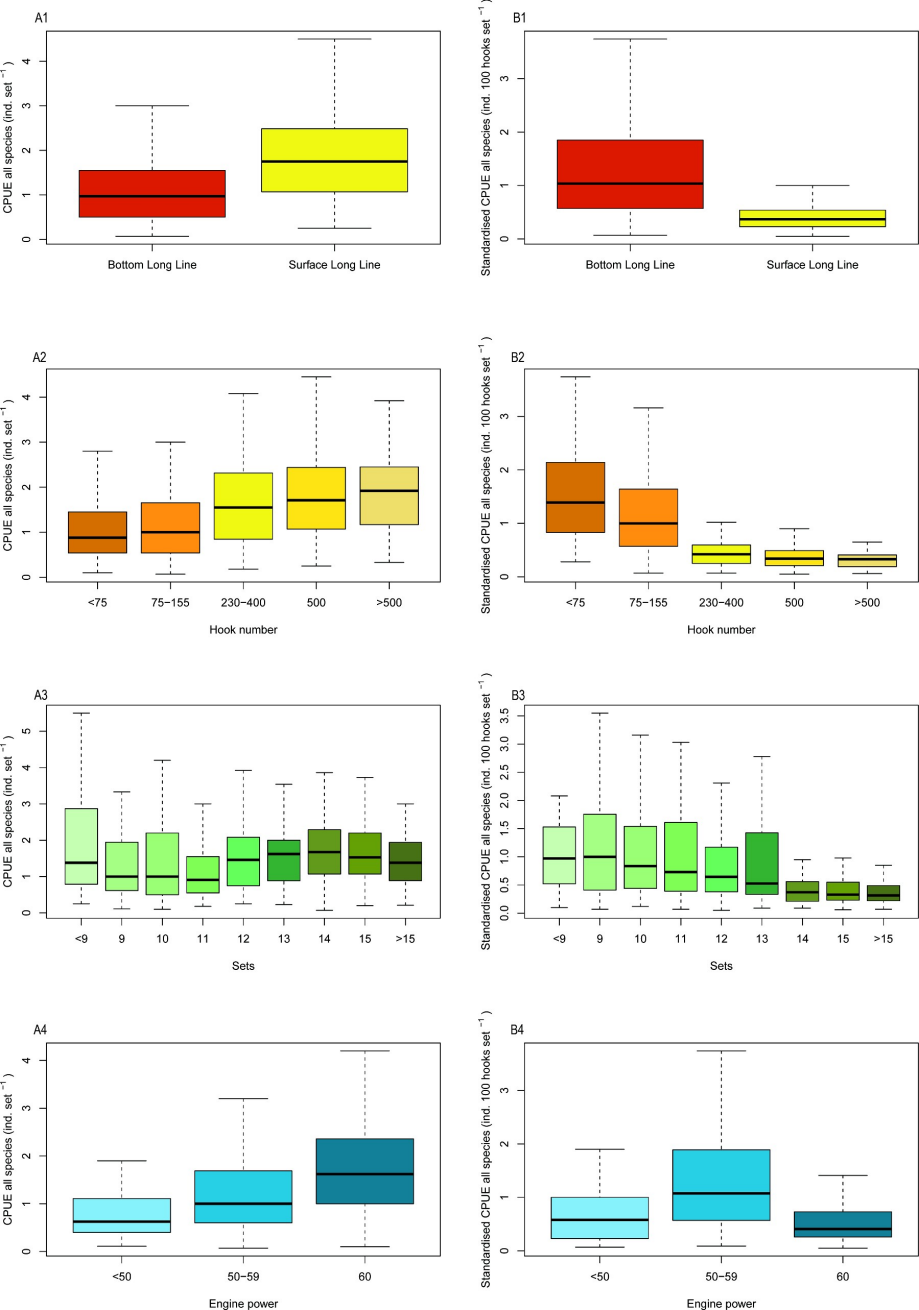
Plots of CPUE: Number of individuals per set (A) and number of individuals per 100 hooks per set (standardised CPUE) (B) by gear type (1), number of hooks (2), number of sets (3) and engine horsepower (4).

Measures of CPUE for the Tanjung Luar shark fishery vary spatially and temporally, and with several aspects of fishing effort including gear type, hook number, engine power and number of sets. An initial comparison of average catch per trip and catch per set of the two major gear types, surface longline and bottom longline, indicates that CPUE of surface longlines was significantly higher than that of bottom longlines (ANOVA, p<0.001). CPUE (individuals per set) was also positively associated with number of hooks, engine power, and number of sets (Fig 2). However, these relationships are for unstandardised CPUE i.e. without controlling for number of hooks.

When controlling for hook number using standardised CPUE (individuals per 100 hooks per set) the relationships were reversed, with standardised CPUE of bottom longlines significantly higher than that of surface longlines (ANOVA, p<0.001; Fig 2). A similar pattern was observed when comparing relationships between CPUE (individuals per set) and standardised CPUE for other measures of fishing effort, including numbers of hooks, engine power and number of sets (Fig 2). There was a positive relationship between unstandardised CPUE (individuals per set) and number of hooks, number of sets and engine power, but a negative relationship between CPUE and these fishing behaviour variables when CPUE was standardised by hook number (individuals per 100 hooks per set).

The best fit LM of standardised CPUE indicated that the most significant factors influencing standardised CPUE were fishing gear and number of hooks (p<0.001). Month, engine power, number of sets and fishing ground were also identified as significant variables (Table 5), although there was considerable covariance between these factors. Standardised CPUE was significantly lower in January, and decreased with higher numbers of hooks, despite a higher total catch per trip and set (Fig 2).

**Table 5:** Analysis of variance for linear model of standardised CPUE (individuals per 100 hooks per set) data from Tanjung Luar; significant values (p<0.05) are given in bold.

	Df	Sum Sq	Mean Sq	F value	P-value
Month	11	4.137	0.376	4.074	9.00*10^**−06**^
Engine power	1	1.612	1.612	17.463	3.39*10^**−05**^
Fishing gear	1	26.500	26.501	287.056	< 2.2* 10^**−16**^
No. hook	1	11.898	11.898	128.881	< 2.2* 10^**−16**^
No. set	1	1.980	1.980	21.443	4.52*10^**−06**^
Fishing ground	2	2.480	1.240	13.432	**2.00.10**^**−06**^
Residuals	568	52	0.0923		

### Factors affecting catch of threatened and regulated species

#### Threatened species

**Fig 3:**
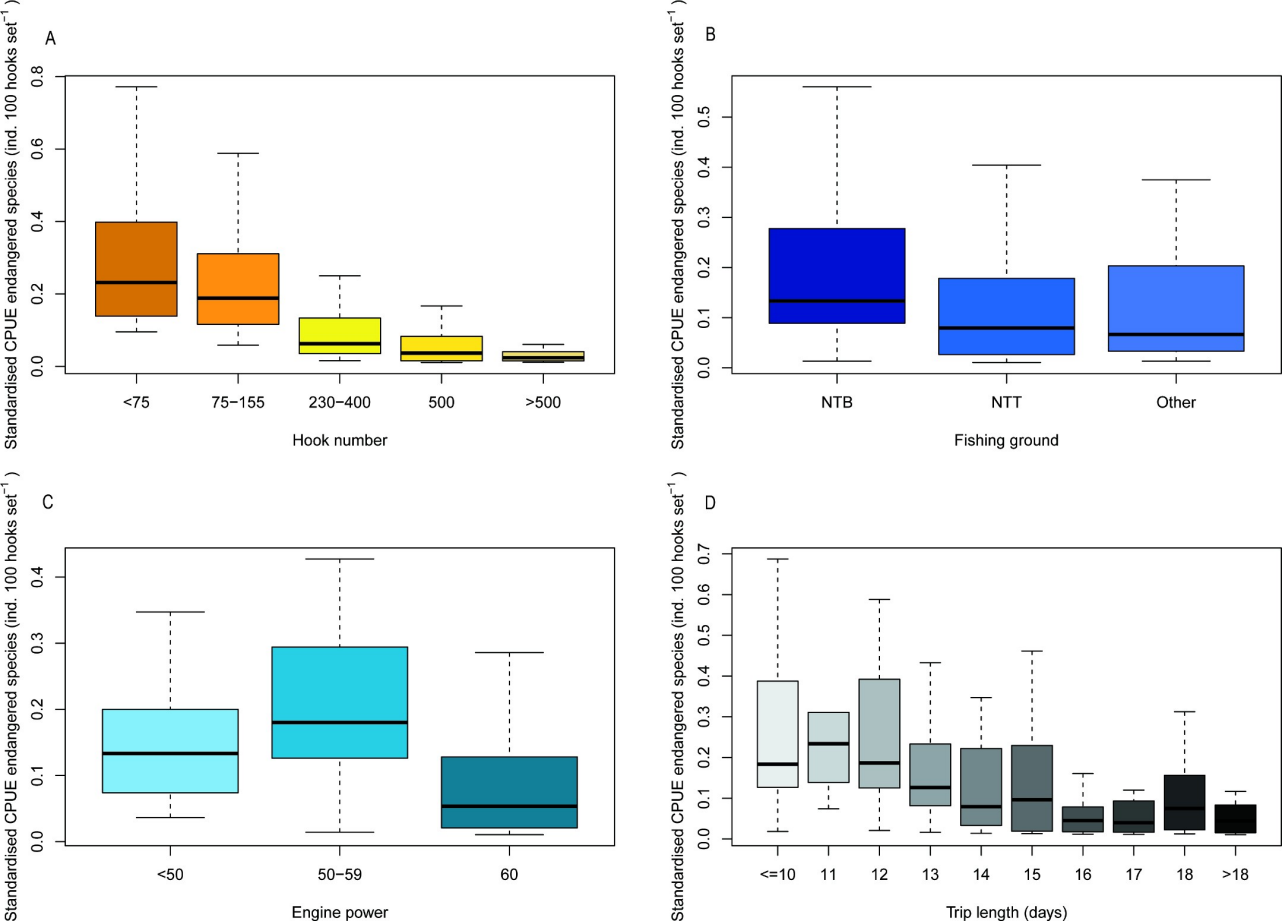
Plots of most significant factors affecting standardised CPUE (number of individuals per 100 hooks per set) of threatened species: a) hook number, b) fishing ground, c) engine power and d) trip length.

Best fit GLMs indicated that the most significant factors influencing the likelihood of catching threatened species were month (January and November were significantly lower: p<0.001 and p<0.05, respectively) and fishing ground (Other (i.e. fishing grounds outside of WNTP and ENTP) was significantly higher: p<0.01). Significant factors associated with standardised CPUE of threatened species were number of hooks (p<0.001), fishing ground (other: p<0.001, ENTP p<0.05), engine power (p<0.001) and trip length (p<0.001) (Table 6 and Fig 3).

**Table 6:** Analysis of variance for the best fit models of factors affecting: a) the likelihood of catching and the standardised CPUE of threatened species b) the likelihood of catching and the standardised CPUE of regulated species.

**a. Fitted model of threatened species**					
**Model: GLM (threatened ~ month + fishing ground, family = binomial)**					
	Df	Deviance Residuals	Df Residuals	Deviance	P-value
NULL		585	797.86		
Month	11	37.149	574	760.72	**0.000109**
Fishing ground	2	12.631	572	748.08	**0.001808**
**Model: LM (Log CPUE threatened ~ month + engine power + no. hook + trip length + no. set + fishing ground)**					
	Df	Sum Sq	Mean Sq	F value	P-value
Month	11	10.188	0.926	7.9919	2.56*10^**−12**^
Engine power	1	6.781	6.781	58.5114	2.38*10^**−13**^
No. hook	1	32.152	32.152	277.4495	<2.2*10^**−16**^
Trip length	1	2.301	2.301	19.8534	1.16*10^**−05**^
No. set	1	0.319	0.319	2.75	0.09823
Fishing ground	2	2.336	1.168	10.0778	5.69*10^**−05**^
Residuals	321	37.199	0.116		
**b. Fitted model of CITES-listed species**					
**Model: GLM(CITES ~ month + engine power + no. hook, family = binomial)**					
	Df	Deviance Residuals	Df Residuals	Deviance	P-value
NULL		585	522.66		
Month	11	53.185	574	469.47	1.66*10^**−07**^
Engine power	1	8.687	573	460.79	**0.003204**
No. hook	1	22.44	572	438.35	2.17*10^**−06**^
**Model: LM(Log CPUE CITES ~ month + engine power + fishing gear + no. hook + no. set + fishing ground)**					
	Df	Sum Sq	Mean Sq	F value	P-value
Month	11	5.003	0.4549	3.1369	**0.000418**
Engine power	1	1.412	1.4125	9.7413	**0.001912**
Fishing gear	1	7.111	7.1107	49.0395	8.68*10^**−12**^
No. hook	1	5.819	5.8192	40.1326	5.54*10^**−10**^
No. set	1	1.679	1.6791	11.5803	**0.000723**
Fishing ground	2	0.529	0.2645	1.8239	0.162525
Residuals	472	68.439	0.145		

#### Regulated species

**Fig 4:**
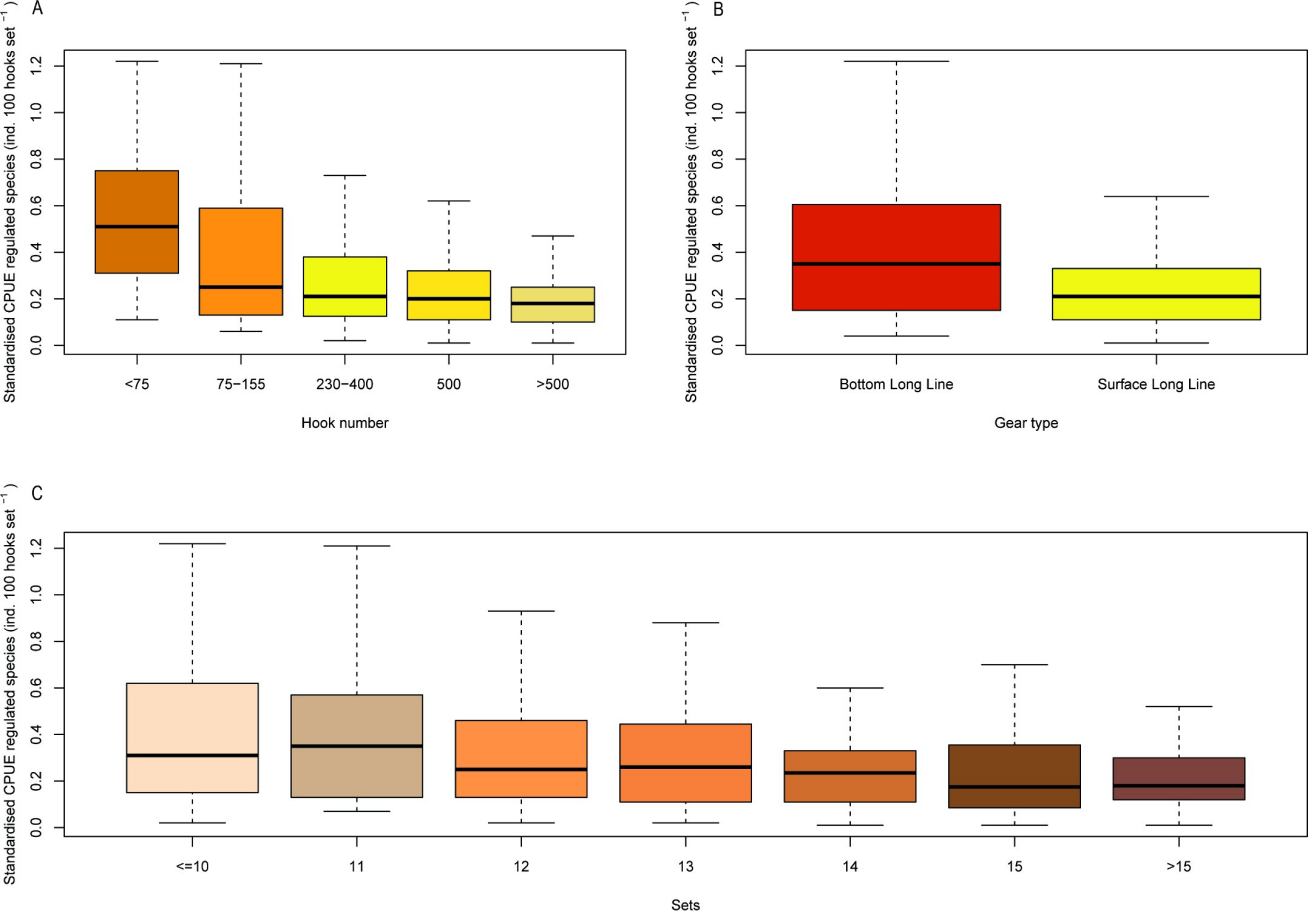
Plots of most significant factors affecting standardised CPUE (number of individuals per 100 hooks per set) of regulated species: a) hook number, b) gear type, c) number of sets.

The most significant factors influencing the likelihood of catching regulated species were month (January was significantly lower: p<0.001), number of hooks (p<0.001) and engine power (<0.01). Significant factors associated with standardised CPUE of regulated species were number of hooks (p<0.001), fishing gear (<0.001), number of sets (p<0.001), engine power (p<0.01) and month (November and January: p<0.05) (Table 5 and Fig 4).

## Discussion

### Catch patterns

Although Tanjung Luar’s targeted shark fishery is small in scale, considerable numbers of shark are landed, including a large proportion of threatened and regulated species. A key finding is that measures of CPUE, for all sharks and for threatened and regulated species, vary spatially and temporally, and with several aspects of fishing effort including gear type, hook number, engine power and number of sets. Moreover, the relationships between CPUE and fishing behaviour variables are different for different measures of CPUE (CPUE per trip, CPUE per set, CPUE per 100 hooks per set). This highlights the importance of using appropriate standardisation for meaningful comparisons of CPUE across different gears and vessel types, and has important implications for fisheries management.

Unstandardised CPUE (individuals per set) was significantly lower in January. This is during the west monsoon season, which is characterised by high rainfall and adverse conditions at sea for fishing. Unstandardised CPUE was also significantly lower in West Nusa Tenggara Province (WNTP) than East Nusa Tenggara Province (ENTP) and other provinces, suggesting a lower abundance of sharks in this area. Engine power had a significant positive influence on unstandardised CPUE, and was also associated with longer trips and more sets, which was likely due to the ability of vessels with larger engines to travel longer distances, over longer time periods, and with higher numbers of sets, to favoured fishing grounds. Unstandardised CPUE was also significantly higher for surface longlines than bottom longlines. However, when standardising CPUE for the number of hooks (i.e. individuals per 100 hooks per set) this relationship was reversed. Bottom longlines exhibit a higher standardised CPUE, with negative relationships between catch per 100 hooks per set and number of hooks and frequency of sets. Vessels with moderate engine horsepower (50-59hp) also had the highest standardised CPUE. Since surface longlines systematically employ significantly more hooks than bottom longlines (400–600 vs 25–200 hooks), and tend to be associated with larger boats, longer trips and more sets, these findings suggest that although increasing fishing effort increased total catch for these gears and trips, there were diminishing returns of this increased effort above low to moderate levels.

A large proportion of Tanjung Luar’s shark catch consisted of threatened (22%) and regulated species (46%). Month is a significant factor in explaining standardised CPUE of both threatened and regulated species, which could indicate seasonal variation in the abundance of these species in the Tanjung Luar fishing grounds, or seasonal impacts on CPUE due to poor weather conditions. Fishing ground was a significant factor in explaining the catch of threatened species but not the catch in regulated species. This may be due to differences in range, distribution and relative abundance of species within these groups. Threatened species make up a relatively small proportion of Tanjung Luar’s catch in comparison to regulated species, which make up almost half of the catch (46%). As such, regulated species may generally be more abundant and spatially diffuse than threatened species, and therefore caught more uniformly across fishing grounds. For example, regulated species catch is dominated by silky sharks (*Carcharhinus falciformis*), which are circum-tropical and coastal-pelagic, and exhibit limited site-fidelity or aggregation behaviour, while threatened species catch is dominated by scalloped hammerheads (*Sphyrna lewini*), which are known to aggregate in schools. These schools of scalloped hammerheads may be more restricted to specific aggregation sites outside of WNTP and ENTP waters, while silky sharks are found in uniform abundance throughout fishing grounds.

As with CPUE of all catch, there was a positive relationship between unstandardised CPUE (catch per set) of threatened and regulated species and number of hooks, but a significant negative relationship between standardised CPUE (catch per 100 hooks per set). This was likely due to diminishing returns of adding additional hooks, and indicates that the effort for threatened and regulated species was exceeding maximum sustainable yield effort, such that increases in effort (e.g. hook number) were leading to decreases in catch [28–30].

### Management implications

Due to the profitability of the shark industry in Tanjung Luar, and limited adaptive capacity and willingness of shark fishers to move into other industries, it is necessary to identify practical and ethical management interventions that can improve the sustainability of the fishery whilst also mitigating the negative socio-economic consequences for coastal communities. Our findings indicate that spatiotemporal closures and restrictions on fishing effort could improve the overall catch per unit effort and sustainability of the Tanjung Luar shark fishery, and lead to positive conservation outcomes for priority species.

Since the location of shark fishing grounds plays a significant role in determining the likelihood of catching threatened species and their associated CPUE, improved marine spatial planning, with the identification of marine protected areas (MPAs) that protect critical shark habitat and shark populations, could reduce catch of species of conservation concern [31–33] and increase abundance of sharks [34, 35]. Provincial governments in West Papua and West Nusa Tenggara have already established ‘shark sanctuary’ MPAs, which protect critical shark habitat and ban shark fishing within their boundaries [16, 36], and monitoring data indicates positive impacts of shark-specific closures on shark abundance [37, 38]. Strengthening Indonesia’s existing MPA network for shark conservation, such as making all MPAs no-take zones for sharks and expanding spatial protection to critical shark habitat, including aggregation sites or pupping and nursery grounds for species of conservation concern, could have considerable conservation benefits. It should be noted, however, that MPAs may only be effective for certain species, such as those with small ranges or site-fidelity [32]. More research is required to identify critical shark habitat and life history stages. For Tanjung Luar these efforts could focus on better understanding scalloped hammerhead (*Sphyrna lewini*) aggregation sites. Well-targeted spatial closures for this species could significantly reduce catch of threatened species in this fishery.

The relationships between gear type, several aspects of fishing effort (i.e. hook number, engine power, number of sets, trip length), standardised CPUE of all shark species and standardised CPUE of threatened and regulated species suggest that there is an optimal effort that could increase overall CPUE of the fishery and significantly reduce fishing mortality of species of conservation concern. For example, our data suggest that CPUE peaks with low to intermediate trip lengths and gear sets, intermediate engine power and hook numbers of less than 75 per set longline. Although standardised CPUE of threatened and regulated species is also higher when fewer hooks are deployed, the catch per set and overall mortality is significantly lower. Regulations that control the number of hooks in combination with incentives for shark fishers to tightly manage the number of hooks they deploy could significantly reduce mortality of threatened and endangered species, maximise the overall CPUE of the fishery, and reduce operational costs for fishers, making shark fishing in Tanjung Luar more sustainable and more cost effective [39–41].

Acknowledging that almost half of Tanjung Luar’s shark catch consists of CITES-listed species, developing measures that ensure both the sustainability of the fishery, and full traceability and control of onward trade, will be crucial for implementing CITES [42]. The Indonesian government has demonstrated a strong commitment to regulating shark trade and implementing CITES [17–18], as demonstrated through several policy decisions to confer full and partial protection to CITES-listed shark and ray species (Marine Affairs and Fisheries Ministerial Decree No 4./KEPMEN-KP/2014, Regulation No. 48/PERMEN-KP/2016). This includes zero quotas/export bans for hammerhead and oceanic whitetip sharks. However, these export bans should be considered intermediate policy measures as monitoring systems and data availability are improved, and sustainable quotas are established. This will be challenging, as shark products are often traded in large volumes of fresh and/or preserved body parts, with high morphological similarity between products from regulated species and non-regulated species. To guarantee that trade is not detrimental to the survival of species, sustainable fisheries management will need to be complemented with species-specific trade quotas. This will require catch documentation systems which trace shark products from point of catch to point of export and rapid, low-cost species identification methods.

As baseline data on shark population health are limited, and there is no standardised, fisheries-independent system for monitoring long-term changes in shark populations, indirect bio-indicators (e.g. endo- and ectoparasites, [43–45]) could help to elucidate the impact of management measures on fisheries and populations of wild species. In the future, shark conservation and fisheries management could benefit from long-term monitoring of agreed indices of population abundance and health status.

These lessons may also apply to shark fisheries in other parts of the world. As sharks increasingly become the focus of global conservation efforts it should be acknowledged that species protection alone will not be enough to reduce mortality of priority species. More needs to be done to identify practical fisheries management measures that can reduce pressure on the most vulnerable species and populations, but also support sustainable use of species that are less susceptible to overfishing. Shark fishing forms an integral part of the livelihood strategies of many coastal communities [22, 23], and prohibiting catches will not necessarily lead to positive conservation outcomes [21, 46]. Management interventions must take into account local context and the motivations and well-being of fisher communities in order to be ethical, feasible and impactful.

## Supporting information

S1 Dataset Data of landed sharks at Tanjung Luar auction that had been used for this study. (XLSX)

S1 File Questionnaires have been used to interview shark fishers, collector, traders, and processors. (DOCX)
