# Supplementary material for: Practical measures for sustainable shark fisheries: Lessons learned from an Indonesian targeted shark fishery
Source: PLoS One. 2018 Nov 2;13(11):e0206437. doi: 10.1371/journal.pone.0206437 (PMC6214517; doi:10.1371/journal.pone.0206437)
Supplement: S1 File — (DOCX) [file pone.0206437.s002.docx]

**Sharks and Rays Fishing and Supply Chain Survey**

**Date __________________**

**Interviewer __________________**

**Respondent No __________________**

**BASIC INFORMATION**

| 1 | Location ID | : | Province : |
| --- | --- | --- | --- |
|  |  |  | District : |
|  |  |  | Sub-district : |
|  |  |  | Village : |
| 2 | Location name | : |  |
| 3 | Fish landing type | : | 1. Fish landing port |
|  |  |  | 1. Fish auction place |
|  |  |  | 1. Fishing village |
|  |  |  | 1. Other (name) :__________________ |
| 4 | Ice factory |  | Is it work: Yes [ ] No [ ] |
| 5 | Cold storage |  | Is it work: Yes [ ] No [ ] |
| 6 | Number of fishing fleet | : |  |
| 7 | Number of shark fishing fleet | : |  |
| 8 | Type of fishing gear | : |  |
| 9 | Type of shark fishing gear | : |  |
| 10 | Number of fisher | : |  |
| 11 | Number of shark fisher | : |  |
| 12 | Number of fish collector | : |  |
| 13 | Number of outside shark fisher | : |  |
| 14 | Number of inside shark fisher | : |  |
| 15 | Fisher group (number and name) | : |  |

**Shark Supply-chain Survey**

FISHER QUESTIONNAIRE

| Date: | Village/hamlet: |
| --- | --- |
| Fisher name: |  |

Location type: □ 1. Community port □2. Fishing village □ 3. □ Fish landing port

**Personal Data**

| Name: | |
| --- | --- |
| Since when you catch shark: | |
| Does your parent also catch shark: Yes [ ] No [ ] | |
| Does your grandfather also catch shark: Yes [ ] No [ ] | |
| Are you member of fisher group/cooperative: Yes [ ] No [ ] What is the name? | |
| Fisher group/cooperative’s name: | |
| Origin : local/native [ ] migrant [ ] non-migrant [ ] | |
| Demographic (stay) status : permanent [ ] seasonal [ ] | |
| Age: | Date of birth: Ethnicity: |
| Status : married [ ] single [ ] | |
| Education level: No school [ ] Elementary school [ ] Junior high school [ ] Senior high school [ ] Bachelor [ ] | |
| Wife’s education level: No school [ ] Elementary school [ ] Junior high school [ ] Senior high school [ ] Bachelor [ ] | |
| Does your wife help you catch/trade shark: Yes [ ] No [ ] | |
| How many people dependence on you : Average monthly expenses: | |

**Operational**

| 5 top most often caught sharks and rays species | species 1: | species 2: | species 3: | species 4: | species 5: |
| --- | --- | --- | --- | --- | --- |
| Fishing gear use (explain it with picture) |  |  |  |  |  |
| Number of hook? |  |  |  |  |  |
| Hook size? |  |  |  |  |  |
| If you are using longline, how deep you put it? Is it bottom longline? | | | Drifting longline |  | Longline length? |
| If you are using net, mention the size?: | | | Number of net? |  |  |
| The smallest sharks and rays ever caught (each species) |  |  |  |  |  |
| Do you have your own gear? (Boat, fishing gear) | | Yes | No |  |  |
| If not, who provide it ? | | Boat? |  | Fishing gear? |  |
| When you are going to fish sharks and rays (month)? | | |  |  |  |
| When is the shark fishing peak season? | | |  |  |  |
| When is the shark fishing low season? | | |  |  |  |
| How many times you fish in a week? | | | trip |  |  |
| How many weeks you fish in one year? | | | week |  |  |
| How long is your fishing trip? | | | hour | day |  |
| How many days you go fishing during shark fishing season?  (peak season) | | |  |  |  |
| How many ice (kg) you bring? | | | Kg |  |  |
| How many fuel you bring? Diesel fuel? Gasoline? | | | Liter |  |  |
| How much the price of fuel? (Rp/Liter) | | | Rp......... |  |  |
| Do you bring other item? Cigarette?  Kerosene? Logistic? Fishing gear?  Bait? Lubricant oil? | | |  |  |  |
|  | | |  |  |  |

| Shark fishing ground :  (How many km from port) |
| --- |
| Does the fishing ground further compare to previous years? Or is it similar for shark fishing ground? |
| Does the shark size smaller, similar, or bigger compare to previous years catches? |
| What bait do you use for fishing shark? |
| Where do you land the shark? |
| Does the shark size smaller, similar, or bigger compare to previous years catches? |
| How many shark fishing boat going when you go fishing shark? |
| In average, how much you get per fishing trip? |
| Why you are specifically fishing for shark? 1. High demand 2. Good price 3. Easily caught 4. Other |
| Beside sharks and rays, have you catch other fish species? |
| What is the percentage of your sharks and rays catch compare to other fish? |
| Which one give bigger profit, fishing sharks and rays or other fish species? |
| Do you have side income other activity (non-fishing)? |
| How much is your income from non-fishing activity? |
| If you are not fishing shark, what would be the source of income if you lost your revenue from shark? |
| How much is roughly the substitute income you will get, and how big is it comparing to your income from shark fishing? |

**Average sharks and rays catch**

| \| No \| Type/species \| Average number of catch  Peak season  (per fish, per kg) \| \| \| Average number of catch  Low season  (per fish, per kg) \| \| \| Average number of catch  Normal day  (per fish, per kg) \| \| \| \| --- \| --- \| --- \| --- \| --- \| --- \| --- \| --- \| --- \| --- \| --- \| \| 10 years ago \| 5 years ago \| Now \| 10 years ago \| 5 years ago \| Now \| 10 years ago \| 5 years ago \| Now \| \|  \|  \|  \|  \|  \|  \|  \|  \|  \|  \|  \| \|  \|  \|  \|  \|  \|  \|  \|  \|  \|  \|  \| \|  \|  \|  \|  \|  \|  \|  \|  \|  \|  \|  \| \|  \|  \|  \|  \|  \|  \|  \|  \|  \|  \|  \| \|  \|  \|  \|  \|  \|  \|  \|  \|  \|  \|  \| \|  \|  \|  \|  \|  \|  \|  \|  \|  \|  \|  \| \|  \|  \|  \|  \|  \|  \|  \|  \|  \|  \|  \| |
| --- | --- | --- | --- | --- | --- | --- | --- | --- | --- | --- | --- | --- | --- | --- | --- | --- | --- | --- | --- | --- | --- | --- | --- | --- | --- | --- | --- | --- | --- | --- | --- | --- | --- | --- | --- | --- | --- | --- | --- | --- | --- | --- | --- | --- | --- | --- | --- | --- | --- | --- | --- | --- | --- | --- | --- | --- | --- | --- | --- | --- | --- | --- | --- | --- | --- | --- | --- | --- | --- | --- | --- | --- | --- | --- | --- | --- | --- | --- | --- | --- | --- | --- | --- | --- | --- | --- | --- | --- | --- | --- | --- | --- | --- | --- | --- | --- | --- |
|  |
| Who buy the sharks and rays you catch?  If the buyer is more than one, mention the percentage/ proportion? |
| Do you sell whole sharks and rays? In parts?  Does the guts cleaned? Only the body? Only the fin? Is it dried? |
| If not whole, who clean it? Cut it? Dry it? |
| If other people do it, how much is the fee to cut, dry it? |
| How much weight loss by cleaning it? Cut it? |
| How much the price of sharks and rays? (per kg or per fish?) |
| - Body? (Rp / 1 fish) - Fin? - Skin? |
| What the buyer do with the sharks and rays you sold? Sell it back? Process it? |
| How many percentage of your catch that you keep or consumed?  If none of it you keep, what kind of fish you bring home? |
| Doe the price of sharks and rays changing within years?  Does it change during Chinese new year? |
| - If there is any change, when the price is the highest?   Why? And how high is the price? |
| - When the price is at the lowest? Why?   How low is the price? |
| In the last 5 years, is there any change on sharks and rays price? Yes or No   - If yes? 2013? - 2012? - 2011? - 2010? - 2009? |
| If the sharks and rays price is changing, do you know the reason why? |
| Does the buyer help to cover operational cost?  (Fuel, bait, maintenance cost) |
| - If yes, is there any requirement that you need to do to be given that benefit?  (e.g. you have to sell your catch only to him/her) |
| - If yes, what is the consequence if you violate the agreement? |

**Management Information**

1. Type of shark fisheries management (these questions should be ask to people whose one of his occupation is shark fisher)

| Management type | Explanation  (Yes/No) | Compliance level; Does people still fishing? | | |
| --- | --- | --- | --- | --- |
|  |  | No | Few | All |
| Is there an area where people are prohibited to catch shark and rays? |  |  |  |  |
| Is there any certain time when people are prohibited to catch shark and rays? |  |  |  |  |
| Is there any particular species of shark and rays that are prohibited to be catched? |  |  |  |  |
| Is there any particular size of shark and rays that are prohibited to be catched? |  |  |  |  |
| Is there any restrictions to the fishing gear that are used? |  |  |  |  |
| Is there any stipulation on the quota of shark and rays’ catch results? |  |  |  |  |

**Knowledge about regulation**

1. Do you think that sharks and rays utilization in Indonesia need to be managed or regulated?
   1. Yes b. No
2. Do you know regulation about sharks and rays in Indonesia?

a. Yes b. No

1. From where do you learn that regulation?
2. Ministry of Marine Affairs and Fisheries
3. Local Government Institution
4. Villager/neighbor/community leader
5. Other source..........
6. Do the community around you accept and support the regulatuion?
7. Not supporting
8. A few/Some supporting the regulation
9. Almost everyone support the regulation
10. Don’t know

**Trade**

| **Boat ownership** | |
| --- | --- |
| a. Work for somebody else’s boat | b. Freelance fisher |
| How long have you work in the recent boat? | Why you become freelance fisher? |
| Boat specification  length:  width:  tonnage:  engine power:  number of crew:  boat registration:  crew: | Have boat? Yes [ ] No [ ]  length:  width:  Engine: Yes [ ] No [ ]  engine power:  boat material: |
| What is your position in the boat?  a. Captain [ ] b. Crew [ ] c. Owner [ ] |  |
| Sharing profit? | Sharing profit? |

Capital

| Buy/build boat | Rp | Boat age? |
| --- | --- | --- |
| Buy boat engine | Rp | Engine age? |
| Buy fishing gear | Rp | Fishing gear age? |
| License | Rp | License period? |
| Boat and engine yearly maintenance | Rp |  |
| Retribution (Daily/monthly/trip) | Rp |  |

**Shark Supply-chain Survey**

COLLECTOR QUESTIONNAIRE

| Date: | Village: |
| --- | --- |
| Name of Collector: |  |

**Personal Data**

| Name of Respondent: | |
| --- | --- |
| Since when did you become shark/ray collector: | |
| Are your parents also shark/ray collectors: Yes [ ] No [ ] | |
| Is your grandfather also a shark/ray collector: Yes [ ] No [ ] | |
| Are you a member of fishermen group/cooperative: Yes [ ] No [ ] What group is it? | |
| Name of fishermen group/cooperative: | |
| Resident : local/indigenous [ ] immigrant [ ] non-immigrant [ ] | |
| What is your residency status: permanent [ ] seasonal [ ] | |
| Age: | Date of birth: Race: |
| Marital Status : married [ ] not-married [ ] | |
| Level of Education: Unschooled [ ] Elementary [ ] Junior High [ ] Senior High [ ] Bachelor [ ] | |
| Wife’s level of education: Unschooled [ ] Elementary [ ] Junior High [ ] Senior High [ ] Bachelor [ ] | |
| Does your wife help in the business: Yes [ ] No [ ] | |
| How many dependents do you have: | |
| How long have you been a collector: | |

**Operational**

| 5 species of sharks and rays you sell often? | Species 1: | Species 2: | Species 3: | Species 4: | Species 5: |
| --- | --- | --- | --- | --- | --- |
| Condition of shark/ray you buy? | Intact? | Dried? | Both |  |  |
| How much do you pay for those sharks and rays? If intact |  |  |  |  |  |
| If not-intact?   - Meat? - Bones? - Fin? - Skin? |  |  |  |  |  |
| How much do you sell those sharks and rays for? |  |  |  |  |  |
| - Meat? - Bones? - Fin? - Skin? |  |  |  |  |  |
| What kind of service do you offer before re-selling those sharks and rays? |  |  |  |  |  |
| - Fins cutting? | Alone? | Other person? Cost? | | How long? | |
| - Intestines cleaning? | Alone? | Other person? Cost? | | How long? | |
| - Skin separation? | Alone? | Other person? Cost? | | How long? | |
| - Drying? | Alone? | Other person? Cost? | | How long? | |
| - Packing? | Alone? | Other person? Cost? | | How long? | |
| - Delivery? | Alone? | Other person? Cost? | | How long? | |
| Where did you buy those sharks/rays from? Explain in detail | | | Fisherman? | Fish auction? | Trader? |
| How many shark and ray you collect in a week? | | | Hiu: | Pari: |  |
| Where do you sell those sharks? (E.g. Mataram? East Lombok? Bali) [If the location is more than one, how much is the percentage of each place?] | | |  | | |
| To whom do you sell the shark/ray? [If the person is more than one, please specify each of the percentage?] | | |  | | |
| Is the price changes within a year? (Ask about Chinese New Year) | | |  | | |
| - If Yes, when is the price is the highest? Why? And how high? - If yes, when is the price is the lowest? Why? And how low? | | |  | | |
| Is the price significantly changes within these past 5 years? Why? | | |  | | |
| - 2013 ? - 2012 ? - 2011 ? - 2010 ? - 2009 ? | | |  | | |
| Is there any opportunity to raise the price? | | |  | | |
| How is the pricing determined? | | |  | | |
| What does the buyer do to the shark/ray you sell? (Re-selling? Where? Processing into another product? Consuming?) | | |  | | |

**Management Information**

1. Type of Management of Shark Fisheries (the question is addressed to a person whose one of the professions is shark fisherman)

| Type of Management | Explanation  (Yes/No) | Compliance level; Does people still fishing? | | |
| --- | --- | --- | --- | --- |
|  |  | No | A few | All |
| Is there an area where people are prohibited to catch shark and rays? |  |  |  |  |
| Is there any certain time when people are prohibited to catch shark and rays? |  |  |  |  |
| Is there any particular species of shark and rays that are prohibited to be catched? |  |  |  |  |
| Is there any particular size of shark and rays that are prohibited to be catched? |  |  |  |  |
| Is there any restrictions to the fishing gear that are used? |  |  |  |  |
| Is there any stipulation on the quota of shark and rays’ catch results? |  |  |  |  |

1. **Knowledge on rules and regulations**
2. Does sharks and rays utilization in Indonesia need to be managed/regulated? What do you think bout this?
   1. Yes b. No
3. Do you know about the regulations related to the shark and ray fisheries in Indonesia?

a. Yes b. No

1. Where did you get that information from?
2. Ministry of Marine Affairs and Fisheries
3. Local Government Institution
4. Villager/neighbor/community leader
5. Other source..........
6. Do the community around you accept and support the regulation?
7. Not supporting
8. A few/Some supporting the regulation
9. Almost everyone support the regulation
10. Don’t know

**Trade**

| Special facilities/building for collector? |  |  |
| --- | --- | --- |
| Number of workers |  | Remuneration system? Profit sharing? |
| Means of shipment? Car? Ship? Plane? |  | Cost? |
| Ice needs? |  |  |
| Retribution (Daily/monthly/trip) where? |  |  |

**Shark Supply-chain Survey**

TRADER QUESTIONNAIRE

| Date: | Village: |
| --- | --- |
| Name of Trader: |  |

**Personal Data**

| Name of Respondent: | |
| --- | --- |
| Since when do you become shark/ray’s trader: | |
| Are your parents also shark/ray’s trader: Yes [ ] No [ ] | |
| Is your grandfather also a shark/ray’s trader: Yes [ ] No [ ] | |
| Are you a member of fishermen group/cooperative: Yes [ ] No [ ] What group is it? | |
| Name of Fishermen Group/Cooperative: | |
| Resident : local/indigenous [ ] immigrant [ ] non-immigrant [ ] | |
| What is your residency status: permanent [ ] seasonal [ ] | |
| Age: | Date of Birth: Race: |
| Marital Status : married [ ] not married [ ] | |
| Level of Education: Unschooled [ ] Elementary [ ] Junior High [ ] Senior High [ ] Bachelor [ ] | |
| Wife’s level of education: Unschooled [ ] Elementary [ ] Junior High [ ] Senior High [ ] Bachelor [ ] | |
| Is your wife/husband helping in trading: Yes [ ] No [ ] | |
| How many dependents do you have: | |
| How long have you been a trader: | |

**Operational**

| 5 species of sharks and rays you sell often? | Species 1: | Species 2: | Species 3: | Species 4: | Species 5: |
| --- | --- | --- | --- | --- | --- |
| Condition of shark/ray you buy? | Intact? | Dried? | Both |  |  |
| How much do you pay for those sharks and rays? If intact |  |  |  |  |  |
| If not-intact?   - Meat? - Bones? - Fin? - Skin? |  |  |  |  |  |
| How much do you sell those sharks and rays for? |  |  |  |  |  |
| - Meat? - Bones? - Fin? - Skin? |  |  |  |  |  |
| What kind of service do you offer before re-selling those sharks and rays? |  |  |  |  |  |
| - Fins cutting? | Alone? | Other person? Cost? | | How long? | |
| - Intestines cleaning? | Alone? | Other person? Cost? | | How long? | |
| - Skin separation? | Alone? | Other person? Cost? | | How long? | |
| - Drying? | Alone? | Other person? Cost? | | How long? | |
| - Packing? | Alone? | Other person? Cost? | | How long? | |
| - Delivery? | Alone? | Other person? Cost? | | How long? | |
| Where did you buy those sharks/rays from? Explain in detail | | | Fisherman? | Fish auction? | Trader? |
| Do you sell those sharks/rays every day? | | | Shark: | Ray: |  |
| Where do you sell those sharks? (E.g. Mataram? East Lombok? Bali) [If the location is more than one, please specify the percentage of each location?] | | |  | | |
| To whom do you sell shark/rays? [If the buyer is more than one, please specify each percentage?] | | |  | | |
| Is there any timeline you propose to the buyer? (if they pay on credit?) | | |  | | |
| IF YES, how long? | | |  | | |
| IF YES, to what kind of buyer? | | |  | | |
| How many collectors are there in the place that you buy shark/rays from? | | |  | | |
| Is trader also cooperating with collector from other area? | | |  | | |
| Where do trader buy their fish? (E.g. at which landing site? At the ocean? Market?) | | |  | | |
| Where do traders sell their fish? (E.g. at which city? [If there are several places at the same time, please specify the percentage of each place?) | | |  | | |
| To whom do traders sell their fish? [If there are more than one person, please specify the percentage of each buyer?] | | |  | | |
| How much is the price of the fish you sell? (IDR/Kg)   - Meat? - Bones? - Fin? - Skin? | | |  | | |
| Is the price changing within a year? (ASK ABOUT PUBLIC HOLIDAY WHICH MAY EFFECTING THE PRICE) | | |  | | |
| - IF YES, when is the time the price becomes most expensive? How much? Why? | | |  | | |
| - **IF YES**, when is the price drops the lowest? How much? Why? | | |  | | |
| Is there any major change to the price within these past 5 years? | | |  | | |
| - If Yes, how much is the price in 2013 - 2012 - 2011 - 2010 - 2009 | | |  | | |
| If yes, in your opinion, why the price of shark and rays changes? | | |  | | |
| Is there any opportunity to raise the price? | | |  | | |
| - IF YES, what is the opportunity? | | |  | | |
| - IF YES, what prevents you from raising the price? | | |  | | |
| - IF YES, what do you need to overcome the problems? | | |  | | |
| What is the chance to increase the quality? | | |  | | |
| - IF YES, what kind of chances is that? | | |  | | |
| - IF YES, what prevents you to increase the quality? | | |  | | |
| - IF YES, what do you need to overcome the problems? | | |  | | |
| What does your buyer do with the fish they buy from you? | | |  | | |
| What is the final destination of those fish? | | |  | | |

**Management Information**

1. Type of Management of Shark Fisheries (the question is addressed to a person whose one of the professions is shark fisherman)

| Type of Management | Explanation  (Yes/No) | Compliance level; Does people still fishing? | | |
| --- | --- | --- | --- | --- |
|  |  | No | A few | All |
| Is there an area where people are prohibited to catch shark and rays? |  |  |  |  |
| Is there any certain time when people are prohibited to catch shark and rays? |  |  |  |  |
| Is there any particular species of shark and rays that are prohibited to be catched? |  |  |  |  |
| Is there any particular size of shark and rays that are prohibited to be catched? |  |  |  |  |
| Is there any restrictions to the fishing gear that are used? |  |  |  |  |
| Is there any stipulation on the quota of shark and rays’ catch results? |  |  |  |  |

**Knowledge on rules and regulations**

1. Does sharks and rays utilization in Indonesia need to be managed/regulated? What do you think about this?
   1. Yes b. No
2. Do you know about the regulations related to the shark and ray fisheries in Indonesia?

a. Yes b. No

1. Where did you get that information from?
2. Ministry of Marine Affairs and Fisheries
3. Local Government Institution
4. Villager/neighbor/community leader
5. Other source..........
6. Do the community around you accept and support the regulatuion?
7. Not supporting
8. A few/Some supporting the regulation
9. Almost everyone support the regulation
10. Don’t know

**Trades**

| Special facilities/building for traders? |  |  |
| --- | --- | --- |
| Number of workers |  | Remuneration system? Profit sharing? |
| Location of sale |  | Cost? |
| Retribution (Daily/monthly/trip) where? |  |  |

**Shark Supply-chain Survey**

PROCESSOR QUESTIONNAIRE

| Date: | Village/hamlet: |
| --- | --- |
| Processor’s name: |  |

**Personal Data**

| Name: | |
| --- | --- |
| Since when you become sharks and rays processor (year): | |
| Does your parents also sharks and rays processors: Yes [ ] No [ ] | |
| Does your grandfather also sharks and rays processors: Yes [ ] No [ ] | |
| Are you member of fisher group/cooperative: Yes [ ] No [ ] What is the name? | |
| Fisher group/cooperative’s name: | |
| Origin : local/native [ ] migrant [ ] non-migrant [ ] | |
| Demographic (stay) status : permanent [ ] seasonal [ ] | |
| Age: | Date of birth: Ethnicity: |
| Status : married [ ] single [ ] | |
| Education level: No school [ ] Elementary school [ ] Junior high school [ ] Senior high school [ ] Bachelor [ ] | |
| Wife’s education level: No school [ ] Elementary school [ ] Junior high school [ ] Senior high school [ ] Bachelor [ ] | |
| Does your husband/wife help you process shark: Yes [ ] No [ ] | |
| How many people dependence on you : | |
| How long you become (fish) processor: | |

**Operational**

| 5 top most often processed sharks and rays species | species 1: | species 2: | species 3: | species 4: | species 5: |
| --- | --- | --- | --- | --- | --- |
| From whom you buy sharks and rays? | 1. Fisher? | | 1. Collector | | 1. Trader in the market? |
| How much you buy the sharks and rays? (Whole) |  |  |  |  |  |
| If not as whole fish?   - Meat? - Bone? - Fin? - Skin? |  |  |  |  |  |
| What is the end product of the sharks and rays you have bought? | 1. Wallet? | 1. Belt? | 1. Shoe? | 1. Oil | 1. other |
| How long does it take, from buying until finish make end product? |  |  |  |  |  |
| **For each product you mentioned earlier**? | | | | | |
| Explain the process of making that sharks and rays products | 1. Wallet? | 1. Belt? | 1. Shoe? | 1. Oil | 1. other |
| Beside material from sharks and rays, is there any other material being use? Mention |  |  |  |  |  |
| - How much is the composition? |  |  |  |  |  |
| - How much it cost? |  |  |  |  |  |
| How much the material depreciated when the product is finished? |  |  |  |  |  |
| How much do you sell to the consumer |  |  |  |  |  |
| Who is your customer?   1. Individual 2. Cooperative 3. Distributor 4. Company 5. Other processor? |  |  |  |  |  |
| From where your customer buy your products?   1. Local (mention) 2. Regional (mention) 3. National 4. International |  |  |  |  |  |
| How consumers get your products?   1. Directly 2. Shipped? (through) |  |  |  |  |  |

**Management Information**

1. Type of shark fisheries management (these questions should be ask to people whose one of his occupation is shark fisher)

| Management type | Explanation  (Yes/No) | Compliance level; Does people still fishing? | | |
| --- | --- | --- | --- | --- |
|  |  | No | Few | All |
| Is there an area where people are prohibited to catch shark and rays? |  |  |  |  |
| Is there any certain time when people are prohibited to catch shark and rays? |  |  |  |  |
| Is there any particular species of shark and rays that are prohibited to be catched? |  |  |  |  |
| Is there any particular size of shark and rays that are prohibited to be catched? |  |  |  |  |
| Is there any restrictions to the fishing gear that are used? |  |  |  |  |
| Is there any stipulation on the quota of shark and rays’ catch results? |  |  |  |  |

**Knowledge about regulation**

1. Do you think that sharks and rays utilization in Indonesia need to be managed or regulated?
   1. Yes b. No
2. Do you know regulation about sharks and rays in Indonesia?

a. Yes b. No

1. From where do you learn that regulation?
2. Ministry of Marine Affairs and Fisheries
3. Local Government Institution
4. Villager/neighbor/community leader
5. Other source..........
6. Do the community around you accept and support the regulatuion?
7. Not supporting
8. A few/Some supporting the regulation
9. Almost everyone support the regulation
10. Don’t know

**Processing**

| Facility/building specifically to process? |  |  |
| --- | --- | --- |
| Number of worker |  | Remuneration system? Sharing profit? |
| Transport mode? Car? Boat? Plane? |  | Cost? |
| Tool/machine use? |  | Price |
| Retribution (Daily/monthly/trip) where? |  |  |
